# Measuring Lower-Limb Kinematics in Walking: Wearable Sensors Achieve Comparable Reliability to Motion Capture Systems and Smartphone Cameras

**DOI:** 10.3390/s25092899

**Published:** 2025-05-04

**Authors:** Peiyu Ma, Qingyao Bian, Jin Min Kim, Khalid Alsayed, Ziyun Ding

**Affiliations:** 1 Dyson School of Design Engineering, Imperial College London, London SW7 2AZ, UK; pm624@ic.ac.uk; 2 School of Engineering, University of Birmingham, Birmingham B15 2TT, UK; kxa066@alumni.bham.ac.uk (K.A.); z.ding@bham.ac.uk (Z.D.); 3 School of Sport, Exercise & Rehabilitation, University of Birmingham, Birmingham B15 2TT, UK; j.m.kim@bham.ac.uk

**Keywords:** inverse kinematics, wearable sensors, smartphone cameras, marker-based, inter-operator reliability

## Abstract

Marker-based, IMU-based (6-axis IMU), and smartphone-based (OpenCap) motion capture methods are commonly used for motion analysis. The accuracy and reliability of these methods are crucial for applications in rehabilitation and sports training. This study compares the accuracy and inter-operator reliability of inverse kinematics (IK) solutions obtained from these methods, aiming to assist researchers in selecting the most appropriate system. For most lower limb inverse kinematics during walking motion, the IMU-based method and OpenCap show comparable accuracy to marker-based methods. The IMU-based method demonstrates higher accuracy in knee angle (5.74 ± 0.80 versus 7.36 ± 3.14 deg, with *p* = 0.020) and ankle angle (7.47 ± 3.91 versus 8.20 ± 3.00 deg, with *p* = 0.011), while OpenCap shows higher accuracy than IMU in pelvis tilt (5.49 ± 2.22 versus 4.28 ± 1.47 deg, with *p* = 0.013), hip adduction (6.10 ± 1.35 versus 4.06 ± 0.78 deg, with *p* = 0.019) and hip rotation (6.09 ± 1.74 versus 4.82 ± 2.30 deg, with *p* = 0.009). The inter-operator reliability of the marker-based method and the IMU-based method shows no significant differences in most motions except for hip adduction (evaluated by the intraclass correlation coefficient-ICC, 0.910 versus 0.511, with *p* = 0.016). In conclusion, for measuring lower-limb kinematics, wearable sensors (6-axis IMUs) achieve comparable accuracy and reliability to the gold standard, marker-based motion capture method, with lower equipment requirements and fewer movement constraints during data acquisition.

## 1. Introduction

Motion capture (Mocap) is a technology used to capture and record the movement of humans or objects. Through the motion capture system, motion data can be obtained in real-time to analyze and reproduce the motion. Mocap technology encompasses a variety of methods for digitally tracking and recording the movement of objects or biological subjects. These methods can be broadly classified into optical-based, inertial-based, electromyography-based, and fusion-based systems [1].

Optical motion capture systems, such as the Vicon system, use infrared cameras to triangulate the position of reflective markers attached to a subject’s body. By attaching markers to the subject’s body and using cameras to capture the movement trajectories of these marker balls, real-time monitoring and analysis of the subject’s posture and actions can be realized. This motion capture method is widely used in motion analysis for improved biomedical insights [2,3,4,5], gait disorder diagnosis [6,7,8], the design and validation of assistive design [9], and other research applications.

An inertial motion capture system is a motion analysis technology based on an inertial measurement unit (IMU) and myoelectric sensor. A commonly used one is the IMU. Inertial sensors provide information captured through multi-axis accelerometers and multi-axis gyroscopes to determine the position and orientation of body segments. Some commercially available inertial sensor designs incorporate multi-axis magnetometers, in which case the sensor orientation is known relative to the world reference frame. Researchers have used varying numbers of inertial sensors to estimate upper limb position and orientation [10,11]. IMUs are more cost-effective than optical motion capture systems, and the motion measurement can be performed without the constraints of site size. 6-axis inertial sensors (accelerometer and gyroscope) and 9-axis inertial sensors (accelerometer, gyroscope, and magnetometer) have issues related to drift [12,13], which is the change in output over time.

Additionally, depth-sensitive cameras measure object distance by projecting light and calculating the time delay between emission and reflection [14,15]. There are also systems that use electromagnetic fields [16] and potentiometers to track the motion of objects, including the relative motion of articulated structures [17]. Furthermore, to improve accuracy and reduce camera occlusion, some hybrid systems combine different Mocap techniques [18].

Different from the marker-based and IMU-based methods, the Stanford research team has developed an open-source real-time motion capture system called OpenCap [19], which captures the movement trajectory and posture of the subject through the built-in camera of the mobile phone. First, it is necessary to collect data from external devices such as IMU and cameras through terminal software such as Vicon Nexus (Version 2.16.0, Vicon Motion Systems Ltd., Oxford, UK) to obtain information such as GRF and marker trajectories. After processing, import OpenSim 4.5 and associate the human body model, and then perform inverse kinematics, inverse dynamics, and other analyses. The advantage of smartphone cameras is that they simplify the hardware requirements and complexity of traditional motion capture systems, make ordinary smartphones a motion capture device, and provide a low-cost, flexible motion analysis solution. Smartphone cameras can complete the scaling and inverse kinematic analysis of the human body online. This method has been used for human motion pattern analysis [20], analysis of the motor ability of patients with limited movement due to illness [21,22], and analysis of sports training performance [23].

Mocap technology has been widely used in various fields due to its diversity. In healthcare and clinical settings, they aid in the diagnosis and treatment of somatic disorders [24]. Mocap technology is also widely used in sports applications to analyze and improve athletes’ postures and improve sports performance [25]. In the industrial field, Mocap technology is mainly used in the entertainment industry [26] and the game industry [27] and has applications in the fields of robotics [28], automobiles [29], and identification of harmful postures at construction sites [30]. In the current research, most scholars use optical Mocap as the gold standard. Table 1 shows the mean absolute error (MAE) on joint angle, joint torque, and ground reaction force (GRF) when smartphone cameras and IMUs are compared with optical Mocap.

In this study, we present the first comparison of the performance of three widely used motion capture methods in gait analysis: the gold standard optical motion capture method and two more economical alternatives, which are the IMU-based method and OpenCap. Our comparison comprehensively includes accuracy and inter-operator reliability, aiming to assess robustness and practical applicability. The goal was to determine which motion capture methods are more reliable, considering the uncertainty introduced by the variability in operators and experimental settings. The experiments in this paper focus on lower limb motion. The motion parameters of the joints listed in Table 2 were studied. The gait information obtained from all motion capture methods was simulated using OpenSim software or related models for a fair comparison. Among them, IMU data were processed using OpenSense workflow, and smartphone data were processed using OpenCap.

## 2. Methodology

### 2.1. Experiment Setup

**Figure 1:**
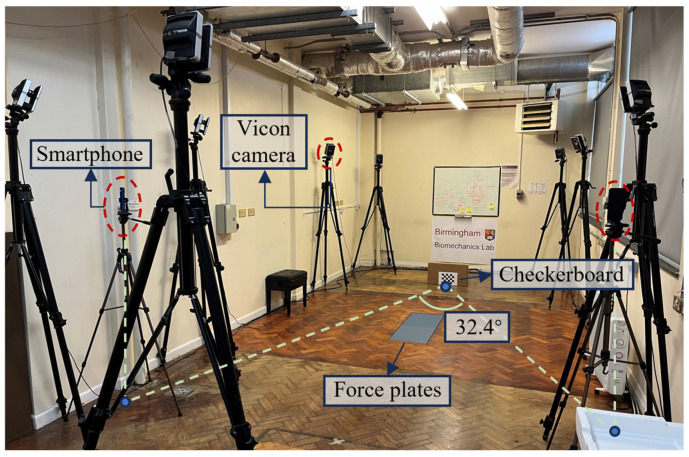
Experimental equipment setup.

**Figure 2:**
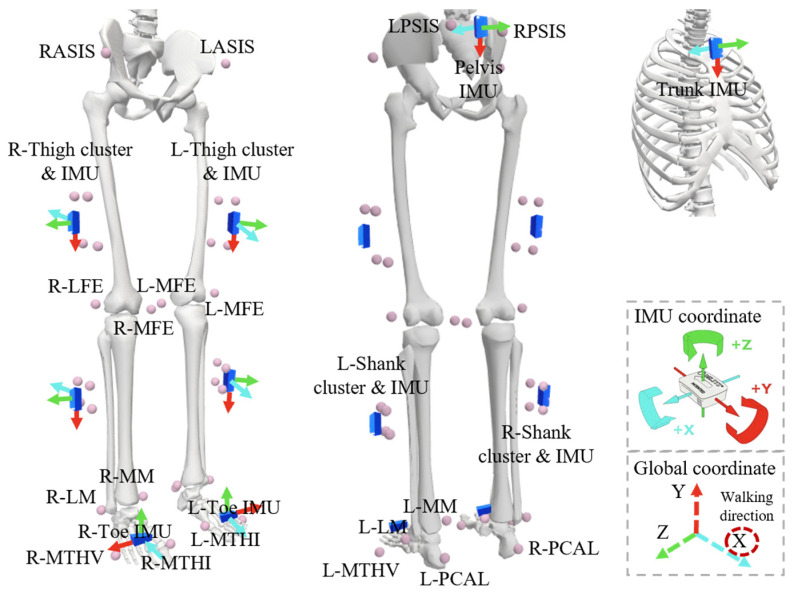
Positions of markers and sensors. Landmarks were placed on the right anterior superior iliac spine (RASIS), left anterior superior iliac spine (LASIS), right posterior superior iliac spine (RPSIS), left posterior superior iliac spine (LPSIS), medial femoral epicondyle (MFE), lateral femoral epicondyle (LFE), right medial malleolus (R−MM), right lateral malleolus (R−LM), left medial malleolus (L−MM), left lateral malleolus (L−LM), right distal first metatarsal (R−MTHI), right fifth metatarsal head (R−MTHV), right posterior calcaneus (R−PCAL), left distal first metatarsal (L−MTHI), left fifth metatarsal head (L−MTHV), left posterior calcaneus (L−PCAL).

In this study, three motion capture methods were used: the marker-based method (Vicon Motion Systems Ltd., Oxford, UK, 100 Hz), the IMU-based method (Delsys Inc., Natick, MA, USA, 2000 Hz), and the smartphone-based method (OpenCap, 60 Hz). OpenCap was used following its standard workflow, including camera calibration with a checkerboard. Two smartphones were symmetrically placed on both sides of the center line of the force plate, with an angle of 32.4° between them and 5 m from the checkerboard. The force plates were used to establish the global coordinate system. Specifically, the Vicon calibration wand was placed at one corner of the force plate at the beginning of the system calibration process. The equipment setup is shown in Figure 1. Eight 6-axis IMUs were attached to the trunk, pelvis, bilateral thighs, shanks, and feet. Forty-two reflective markers were placed on the anterior/posterior superior iliac spine, medial/lateral femoral epicondyles, medial/lateral malleoli, second/fifth metatarsal head, posterior calcaneus, four clusters (with four markers on each cluster) on the lateral aspect of the thigh and shank and 8 IMUs. The arrows in Figure 2 indicate the approximate direction of the IMUs. The +Z-axis of the trunk IMU, the +X-axis of the three IMUs on the left leg and foot, the -X-axis of the three IMUs on the right leg and foot, and the −Z-axis of the pelvis IMU are roughly facing the direction of movement.

We had two operators, one expert with extensive experience in palpating bony landmarks and one novice, who had received only a single training session and was conducting the experiment for the first time. Both operators conducted the marker-based method and IMU-based method for all subjects, while the expert operator set up the OpenCap system. Prior to the start of data acquisition, subjects completed a short practice session walking at a cadence of 110 bpm to familiarise themselves with the task in the lab. Each formal trial involved walking approximately 7 m, containing 7 to 10 gait cycles. For data analysis, we used one gait cycle from each trial, specifically the one in which the participant stepped on the force plates. This gait cycle was chosen for its optimal marker visibility and the participant’s stable walking speed. Each subject performed 6 walking trials, wearing both IMU sensors and markers [10,21,22,35]. Additionally, 3 trials of a neutral standing posture (static posture) were recorded before the dynamic walking trials to allow for IMU zero-drift calibration. After completing the walking trials, the sensors and markers were removed. The second operator then repeated the identical protocol for data acquisition. The order of the operators was randomized. In total, each subject performed approximately 12 walking trials, with a break provided when requested.

Seven healthy male subjects with no self-reported lower limb musculoskeletal pain or impairments were recruited. Institutional ethics approval (Approval No. aer_ERN_21-0519) and informed consent were obtained. The reason for selecting all male subjects is that there are differences in biomechanical characteristics between men and women. To reduce the interference caused by gender differences, only male subjects were selected in this experiment. Each subject recorded their age, height, and weight, as shown in Table 3.

### 2.2. Data Processing

For the marker-based method, we used Vicon Nexus (Version 2.16.0, Vicon Motion Systems Ltd., Oxford, UK) for data acquisition and preliminary processing, including marker labeling, gap filling for missing data, and heel strike detection (using the vertical component of the posterior calcaneus marker [36]). The motion between two heel strikes was considered as one walking period. The heel strikes were also used for data alignment between three motion capture methods. In OpenSim, the marker trajectory file of the static posture exported by Vicon was matched with the virtual marker points in the scaled model, and then the model was scaled using the scale tool. The required kinematic solution was obtained by performing the inverse kinematics tool by using the trajectory file of the walking motion based on the scaled model.

**Figure 3:**
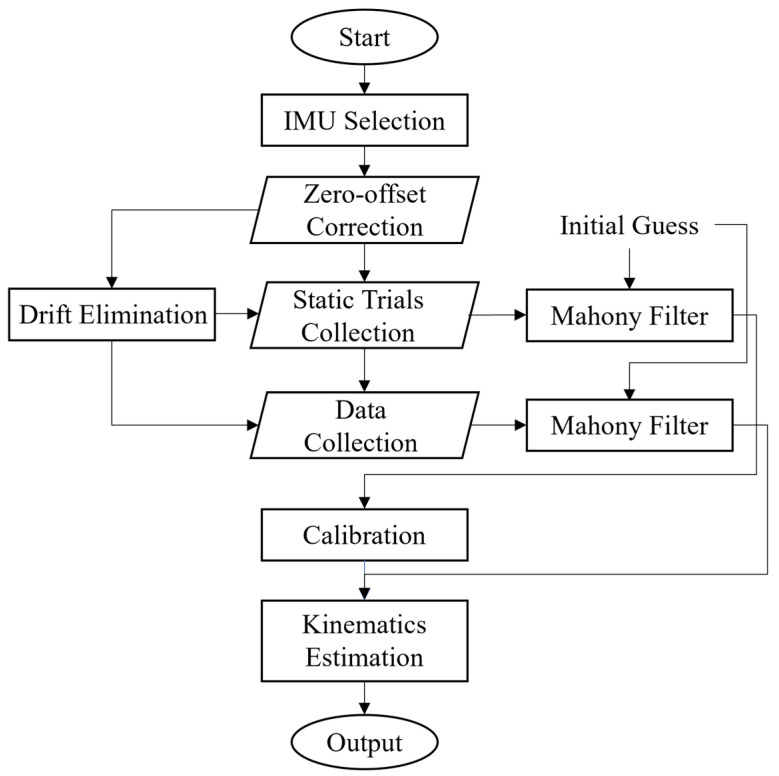
Data analysis flowchart of IMU-based method.

Figure 3 illustrates a data processing pipeline of the IMU-based method, aiming to achieve precise kinematics estimation. The process begins with the selection of 6-axis IMU sensors (i.e., Delsys Inc., Natick, MA, USA). Then, the sensors are placed horizontally with the +*Z*-axis facing upward for zero-offset correction and initial calibration, aiming at eliminating static bias in the accelerometer. Following this, static and dynamic walking trial data are collected, and a sensor fusion algorithm is performed using the Mahony filter. This filter integrates accelerometer and gyroscope data through a proportional-integral feedback mechanism, effectively suppressing gyroscope drift and high-frequency noise from the accelerometer, and outputs the orientation of each sensor in quaternion form. The estimated orientation data are formatted into OpenSim-compatible files and fused with data in the static posture to support accurate model calibration. Subsequently, the IMU Placer tool in OpenSim is used to align sensor frames with the musculoskeletal model, and the IMU Inverse Kinematics tool is applied to compute joint angles by minimizing the orientation discrepancies between model-based virtual IMU and measured IMU data. This pipeline, implemented using MATLAB (Version R2024a, The MathWorks, Inc., Natick, MA, USA), encompasses the complete steps from data acquisition to result output, providing a reliable data foundation for kinematic analysis [35].

OpenCap analyzed and processed the captured videos online to obtain the scaled model, key joint motion trajectory, and inverse kinematics results, which can be directly used for comparison and analysis with other methods.

The sampling frequencies are 100 Hz, 2000 Hz, and 60 Hz for Vicon, IMUs, and OpenCap, respectively. To ensure fair comparison across the three measurement methods with different sampling frequencies, the gait cycle extracted from each trial was resampled to 101 data points using linear interpolation. This normalization enables temporal alignment across methods, facilitating subsequent statistical analysis and comparison.

### 2.3. Data Analysis

**Figure 4:**
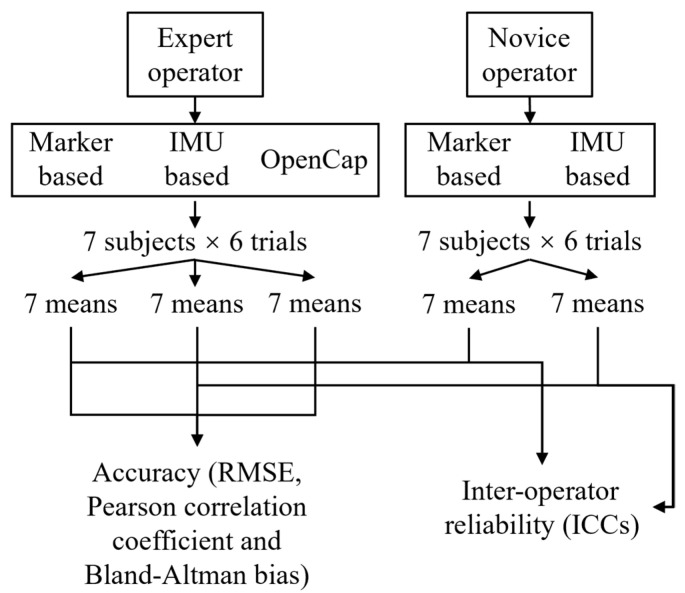
Data analysis flowchart.

The lower limb kinematics obtained using the IMU-based method and OpenCap were evaluated based on accuracy and inter-operator reliability. For the motion capture methods, heel strikes were used to extract data from the same walking gait with 101 data points. The mean was obtained by averaging the data obtained from the 6 trials of each subject under the same motion capture method. For the operators, 7 sets of mean were obtained in the experiment of each motion capture method. The accuracy of different methods was compared using the data from the expert operator. The IMU-based method and OpenCap were compared against the marker-based method’s kinematic results as the gold standard. Accuracy was assessed using the root mean square error (RMSE), Pearson correlation coefficient (*r*), and Bland—Altman bias (B). The inter-operator reliability of the IMU-based method and OpenCap was evaluated using the intraclass correlation coefficient (ICC), two-way random-effects model, and absolute agreement model, where values less than 0.5, between 0.5 and 0.75, between 0.75 and 0.90, and greater than 0.90 indicate poor, moderate, good, and excellent reproducibility, respectively [37]. The ICC for each joint of each subject was calculated (as the mean of the ICC for 200 data points) along with the range of motion (ROM) during the gait cycle, and the results were expressed as the mean over 7 subjects. Figure 4 shows the flowchart of the data analysis in the study.

## 3. Results

### 3.1. Accuracy Comparison

Compared to the marker-based method, the IMU-based method showed significantly better accuracy in knee angle and ankle angle, while OpenCap showed significant accuracy in pelvis tilt, hip adduction, and hip rotation. This was evaluated through RMSE (5.74 ± 0.80 versus 7.36 ± 3.14 deg in knee angle; 7.47 ± 3.91 versus 8.20 ± 3.00 deg in ankle angle; 5.49 ± 2.22 versus 4.28 ± 1.47 deg in pelvis tilt; 6.10 ± 1.35 versus 4.06 ± 0.78 deg in hip adduction; 6.09 ± 1.74 versus 4.82 ± 2.30 deg in hip rotation), the *p*-value of RMSE (0.020 in knee angle; 0.011 in ankle angle; 0.013 in pelvis tilt; 0.019 in hip adduction; 0.009 in hip rotation), and Bland—Altman bias ([1.94, 5.86] versus [−2.92, 4.66] deg in knee angle; [−3.77, 11.23] versus [1.12, 10.56] deg in ankle angle; [−5.30, 8.51] versus [−4.13, 6.31] deg in pelvis tilt; [−3.67, 7.52] versus [−3.17, 3.55] deg in hip adduction; [0.04, 7.29] versus [−1.57, 8.60] deg in hip rotation). Compared to the marker-based, IMU-based method exhibited better accuracy than OpenCap in pelvis list, hip flexion, and hip rotation, but with no significant differences in RMSE and small differences in the range of Bland—Altman bias. The IMU-based method and OpenCap both demonstrate good repeatability in pelvis rotation, hip flexion, knee angle, and ankle angle. However, the repeatability of both methods was poor for pelvis tilt and pelvis list. The OpenCap exhibited better repeatability in hip adduction and hip rotation, which is reflected in the Pearson correlation coefficient *r* (0.59 versus 0.89 in hip adduction; 0.33 versus 0.73 in hip rotation). The above data can be obtained from Table 4.

**Figure 5:**
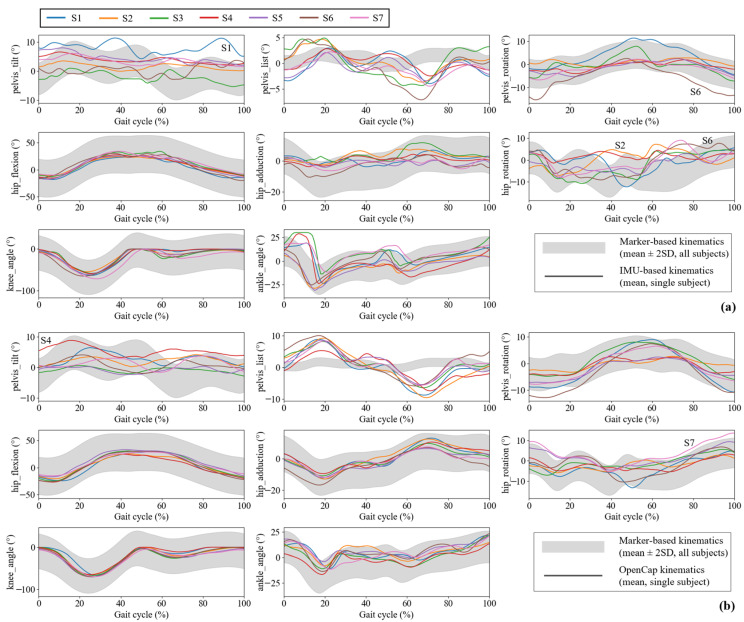
Lower limb kinematics for IMU-based method and OpenCap. Individual subjects’ IMU-based (**a**) and OpenCap (**b**) kinematics for the right side of the body during one overground walking period (7 subjects, mean for 6 trials) are shown as lines. Mean ± 2 standard deviations (SD) for optical-based kinematics are shown as a grey-shaded band.

Figure 5 shows the average joint angles obtained from the IMU-based method and OpenCap for seven subjects across six trials. The IMU-based method and OpenCap data for most joint angles showed better accuracy (within two standard deviations) compared to optical motion capture data, with good consistency across different subjects. However, the pelvic list showed poor agreement on both two methods, and the data of a few subjects (S1, S2, and S6 in the IMU-based method; S4 and S7 in OpenCap) fell outside the two standard deviation range for pelvis tile or hip rotation.

### 3.2. Inter-Operator Comparison

Table 5 shows the ICC of the average experimental data for each degree of freedom obtained by two operators using the marker-based and IMU-based methods for seven subjects. Each ICC is based on a total of 200 data points from both operators. The ICC values for the marker-based method range from [0.001, 0.994], while the ICC values for the IMU-based method range from [0.079, 0.998]. Comparing the mean values across all subjects for each joint, the IMU-based method improves inter-operator reliability in pelvis tilt (0.129 versus 0.354, *p* = 0.156), pelvis list (0.710 versus 0.753, *p* = 0.578), hip flexion (0.917 versus 0.947, *p* = 0.375) and hip rotation (0.577 versus 0.710, with *p* = 0.203), but these differences are not statistically significant. However, the marker-based method demonstrates significantly better inter-operator reliability than the IMU-based method in hip adduction (0.910 versus 0.511, *p* = 0.016).

**Figure 6:**
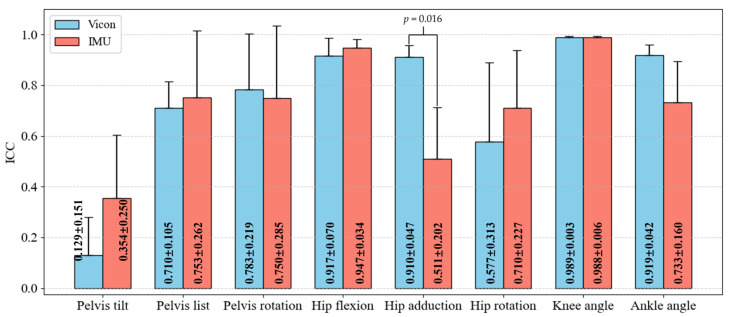
Reliability (ICC) Comparison: Vicon vs. IMU (Mean ± SD).

Figure 6 presents a comparison of the mean inter-operator reliability across subjects between marker-based and IMU-based methods. The ICC value for the marker-based method was significantly higher for hip adduction compared to the IMU-based method; however, no differences were observed in other kinematics variables. Both methods showed low reliability for pelvis tilt, with high variability across subjects, indicating that the current marker and IMU placement protocols (limited to the lower limbs) are insufficient for accurately measuring pelvis tilt motion.

## 4. Discussion

This study demonstrates that both the IMU-based method and OpenCap are reliable alternatives to the marker-based motion capture method for lower-limb kinematics, with the IMU-based method excelling in accuracy for joints with larger motion ranges and OpenCap performing better for joints with smaller motion ranges.

Compared with OpenCap, the IMU-based method showed superior accuracy in knee angle and ankle angle. OpenCap demonstrated better accuracy in pelvis tilt, hip adduction, and hip rotation, as supported by lower RMSE values and similar Bland—Altman biases. Notably, although OpenCap exhibited better accuracy in these parameters, the *p*-values of the non-parametric Wilcoxon signed-rank test between OpenCap and the IMU-based method are not statistically significant. Both methods exhibited good repeatability in pelvis rotation, hip flexion, knee angle, and ankle angle, suggesting they can consistently measure these joint movements. However, the pelvis tilt and pelvis list showed poor repeatability in both methods, indicating a need for improvement in tracking this degree of freedom for lower limb inverse kinematics during walking. These findings indicate that both the IMU-based method and OpenCap are reliable for inverse kinematics during walking motion, with the IMU-based method having higher accuracy for joints with large motion ranges and OpenCap having better accuracy for joints with small motion ranges. Inter-operator reliability analysis showed that, except for hip adduction (*p* = 0.016), the operator reliability of the IMU-based method for other movements is not significantly different from that of the marker-based method.

Both methods exhibited poor data consistency and repeatability in pelvis tilt, with data from some subjects exceeding the two-standard deviation range of the gold standard mean. This may be due to the subtle variations in pelvis tilt during walking.

In addition to evaluating accuracy and inter-operator reliability, we further considered each method’s practical characteristics—including equipment cost, setup complexity, environmental constraints, and portability—to explore their potential for broader clinical or field-based applications. The Vicon system, while offering high precision, relies on expensive, non-portable equipment. Marker placement (with at least three markers per segment and even more for kinematic optimization), calibration, and data processing are time-consuming and require operators with substantial anatomical knowledge. As such, it is best suited for use in controlled laboratory environments. IMUs, as wearable sensors, offer greater convenience, superior portability, and faster setup compared to the Vicon system. They also provide more user-friendly data processing for non-experts and do not require anatomical expertise for use. Additionally, IMUs are not constrained by optical tracking requirements or camera positioning and are relatively low in cost. Unlike OpenCap, which depends on fixed camera placements and is limited by line-of-sight and field-of-view restrictions, IMUs can be deployed in more flexible and large-scale environments and do not require internet connectivity for data processing. Compared to a marker-based method and OpenCap, the IMU-based method demonstrates significant potential for outdoor applications, remote assessments, and data collection in resource-limited settings.

The wearable sensors (6-axis IMUs) achieve comparable reliability and accuracy to other motion capture methods (Vicon, OpenCap) in measuring lower-limb kinematics, but their performance still holds potential for further improvement due to inherent limitations of the sensors. In this study, 6-axis IMUs comprising an accelerometer and a gyroscope were utilized, relying on the initial pose and sensor placement for heading correction. However, 6-axis IMUs exhibit limitations in determining the heading angle: the accelerometer cannot directly measure the heading angle and is susceptible to interference from motion-induced accelerations during dynamic movements, while the gyroscope suffers from integration drift and lacks an absolute reference, causing the heading angle estimation to deviate from the true value over time. In contrast, magnetometers provide absolute heading angle information, enabling the correction of gyroscope drift and enhancing the precision of orientation estimation. Therefore, the adoption of a 9-axis IMU incorporating a magnetometer represents a feasible approach to further improve the accuracy of the IMU-based motion capture method proposed in this study.

This study is an exploratory experiment that only included healthy young male participants. The results may not be directly generalized to older adults, females, or individuals with gait abnormalities. Therefore, future studies should include more diverse participant groups to verify whether the findings of this study have broader applicability. Moreover, the study did not perform multiple comparison corrections; there may be statistical biases. Future studies could apply more rigorous statistical methods to ensure the robustness and reliability of the results. Nevertheless, since each participant contributed a large amount of data, the study still offers useful insight into the initial evaluation of method reliability.

## 5. Conclusions

This study highlights the potential of wearable sensors to provide reliable and efficient motion capture in practical applications. Both the IMU-based method and OpenCap are reliable alternatives to the marker-based motion capture method for lower-limb kinematics measurement. The IMU-based method can be used confidently for common non-pathological gait parameters (knee angle, ankle angle with a large range of motion), while the interpretation of joint parameters with a small range of motion (pelvis tilt, hip adduction, and hip rotation) should be more cautious. In addition, the IMU-based method showed comparable repeatability to OpenCap and comparable inter-operator reliability to traditional marker-based methods. With lower equipment requirements and fewer sensor placement constraints, the IMU-based method has the potential to become an ideal solution for large-scale, long-distance motion data collection outside the laboratory.

## Figures and Tables

**Table 1:** Smartphone cameras and IMUs compared with optical Mocap (BW-body weight, BH-body height).

Motion Capture Method	Joint Angle MAE (deg)	Joint Torque MAE (%BW×BH)	GRF MAE (%BW)
IMU	3~7 [31,32,33]	0.6–1.4 [34]	12.4% [33]
Smartphone cameras [19]	4.5	0.3–1.7	1.5–11.1%

**Table 2:** Comparison of motion parameters in this study.

Joint/Segment	Direction of Movement
Pelvis	Tilt
	List
	Rotation
Knee	Flexion
Ankle	Flexion
Hip	Flexion
	Adduction
	Rotation

**Table 3:** Information of subjects (BMI: body mass index).

No.	Age	Height (cm)	Weight (kg)	BMI (kg/m^2^)
1	21	161.5	79.7	30.56
2	38	172.0	65.4	22.11
3	23	176.3	102.1	32.85
4	29	170.0	51.1	17.68
5	22	181.8	80.1	24.24
6	23	179.5	67.8	21.04
7	21	176.6	79.8	25.59

**Table 4:** The accuracy of lower limb kinematics measured using the IMU-based method and OpenCap during walking gait was compared with the marker-based kinematics. Bland–Altman bias (B) is expressed as ranges, while RMSE and the Pearson correlation coefficient (*r*) are presented as mean across seven subjects. A non-parametric Wilcoxon signed-rank test was performed to compare the differences in RMSE between the two methods. (^a^ indicates significantly different (*p* ≤ 0.05)).

	**RMSE**	***r***	**B**	**RMSE**	***r***	**B**
	Pelvis Tilt			Pelvis List		
IMU	5.49 (2.22)	0.10	−5.30, 8.51	3.86 (1.52)	0.08	−5.12, 4.22
OpenCap	4.28 (1.47)	0.26	−4.13, 6.31	5.35 (0.97)	0.10	−4.57, 3.81
*p*-value	0.013 ^a^			0.125		
	Pelvis rotation			Hip flexion		
IMU	2.89 (1.75)	0.85	−2.17, 5.89	5.16 (1.32)	0.99	−5.77, 5.02
OpenCap	2.81 (1.41)	0.88	−3.25, 1.37	6.16 (2.79)	0.97	−7.45, 1.50
*p*-value	0.306			0.200		
	Hip adduction			Hip rotation		
IMU	6.10 (1.35)	0.59	−3.67, 7.52	6.09 (1.74)	0.33	0.04, 7.29
OpenCap	4.06 (0.78)	0.89	−3.17, 3.55	4.82 (2.30)	0.73	−1.57, 8.60
*p*-value	0.019 ^a^			0.009 ^a^		
	Knee angle			Ankle angle		
IMU	5.74 (0.80)	0.99	1.94, 5.86	7.47 (3.91)	0.80	−3.77, 11.23
OpenCap	7.36 (3.14)	0.94	−2.92, 4.66	8.20 (3.00)	0.80	1.12, 10.56
*p*-value	0.020 ^a^			0.011 ^a^		

**Table 5:** Inter-operator reliability in measuring lower limb kinematics using the marker-based method and IMU-based method was compared during one walking gait. ICCs were calculated for the range of motion (ROM) and the whole gait cycle of the subjects using the mean results of six trials. A non-parametric Wilcoxon signed-rank test was performed to compare the differences in ICCs of the subjects between the two methods. (^a^ indicates significantly different (*p* ≤ 0.05)).

	ICC							
	Pelvis tilt		Pelvis list		Pelvis rotation		Hip flexion	
	Vicon	IMU	Vicon	IMU	Vicon	IMU	Vicon	IMU
ROM	0.194	0.529	0.563	0.793	0.283	0.964	0.641	0.931
S1	0.095	0.302	0.702	0.886	0.528	0.753	0.761	0.902
S2	0.472	0.154	0.560	0.133	0.374	0.079	0.951	0.912
S3	0.066	0.082	0.802	0.890	0.966	0.942	0.988	0.928
S4	0.001	0.834	0.783	0.903	0.933	0.938	0.933	0.999
S5	0.045	0.134	0.557	0.946	0.959	0.805	0.969	0.948
S6	0.035	0.545	0.839	0.745	0.912	0.960	0.891	0.986
S7	0.192	0.429	0.726	0.765	0.809	0.773	0.927	0.953
Mean	0.129	0.354	0.710	0.753	0.783	0.750	0.917	0.947
*p*-value	0.156		0.578		0.688		0.375	
	Hip adduction		Hip rotation		Knee angle		Ankle angle	
	Vicon	IMU	Vicon	IMU	Vicon	IMU	Vicon	IMU
ROM	0.653	0.749	0.082	0.840	0.883	0.885	0.585	0.026
S1	0.916	0.603	0.304	0.891	0.990	0.984	0.914	0.746
S2	0.978	0.218	0.091	0.842	0.991	0.980	0.829	0.876
S3	0.936	0.756	0.933	0.935	0.983	0.988	0.923	0.934
S4	0.837	0.534	0.942	0.302	0.992	0.996	0.926	0.554
S5	0.943	0.243	0.339	0.438	0.994	0.998	0.929	0.456
S6	0.849	0.749	0.811	0.847	0.986	0.988	0.983	0.830
S7	0.914	0.473	0.619	0.718	0.989	0.987	0.926	0.736
Mean	0.910	0.511	0.577	0.710	0.989	0.988	0.919	0.733
*p*-value	0.016 ^a^		0.203		0.984		0.078	

## Data Availability

The data presented in this study is available on request from the corresponding author.
